# Simultaneous modulation of the intrinsic and extrinsic pathways by simvastatin in mediating prostate cancer cell apoptosis

**DOI:** 10.1186/1471-2407-12-409

**Published:** 2012-09-14

**Authors:** Anna Goc, Samith T Kochuparambil, Belal Al-Husein, Ahmad Al-Azayzih, Shuaib Mohammad, Payaningal R Somanath

**Affiliations:** 1 Clinical and Experimental Therapeutics, College of Pharmacy, University of Georgia, Augusta, GA, USA; 2 Charlie Norwood VA Medical Center, Augusta, GA, USA; 3 Department of Medicine, Georgia Health Sciences University, Augusta, GA, USA; 4 Clinical and Experimental Therapeutics, College of Pharmacy, University of Georgia, HM1200 – Georgia Health Sciences University, Augusta, GA, 30912, USA

**Keywords:** Prostate cancer, Simvastatin, Docetaxel, Apoptosis, Bcl-2, Fas-L

## Abstract

**Background:**

Recent studies suggest the potential benefits of statins as anti-cancer agents. Mechanisms by which statins induce apoptosis in cancer cells are not clear. We previously showed that simvastatin inhibit prostate cancer cell functions and tumor growth. Molecular mechanisms by which simvastatin induce apoptosis in prostate cancer cells is not completely understood.

**Methods:**

Effect of simvastatin on PC3 cell apoptosis was compared with docetaxel using apoptosis, TUNEL and trypan blue viability assays. Protein expression of major candidates of the intrinsic pathway downstream of simvastatin-mediated Akt inactivation was analyzed. Gene arrays and western analysis of PC3 cells and tumor lysates were performed to identify the candidate genes mediating extrinsic apoptosis pathway by simvastatin.

**Results:**

Data indicated that simvastatin inhibited intrinsic cell survival pathway in PC3 cells by enhancing phosphorylation of Bad, reducing the protein expression of Bcl-2, Bcl-xL and cleaved caspases 9/3. Over-expression of PC3 cells with Bcl-2 or DN-caspase 9 did not rescue the simvastatin-induced apoptosis. Simvastatin treatment resulted in increased mRNA and protein expression of molecules such as TNF, Fas-L, Traf1 and cleaved caspase 8, major mediators of intrinsic apoptosis pathway and reduced protein levels of pro-survival genes Lhx4 and Nme5.

**Conclusions:**

Our study provides the first report that simvastatin simultaneously modulates intrinsic and extrinsic pathways in the regulation of prostate cancer cell apoptosis *in vitro* and *in vivo*, and render reasonable optimism that statins could become an attractive anti-cancer agent.

## Background

Statins, the cholesterol lowering drugs, are some of the most commonly prescribed medications. Recently, attention has focused on the development of statins as therapeutic agents for the treatment of solid and hematological cancers [1]. Statins elicit pleiotropic effects on various cell types and differentially modulate cellular functions such as cell migration, proliferation, cell survival and apoptosis in normal and malignant cells [2]. Lipophilicity, dose and duration of the treatment as well as cell type are all determining factors on the specific effect of a statin on the outcome of a cell function. According to the American Cancer Society, prostate cancer is the most commonly diagnosed cancer and the second leading cause of cancer death in American men. Many recent clinical studies have indicated that use of statins is associated with >50% reduction in prostate cancer deaths [3,4]. Our previous study showed that simvastatin, a lipophilic statin inhibited multiple prostate cancer cell functions *in vitro* such as migration, proliferation, cell survival and colony formation as well as tumor growth in a nude mouse xenograft *in vivo*, mainly via inhibition of Akt pathway [5]. However, exact molecular mechanisms by which statins modulate each of the prostate cancer cell function are not clear.

One of the factors that determine the efficacy of a cancer drug is its ability to inhibit cancer cell survival and induce apoptosis. Meantime, a major concern over the use of anti-cancer drugs for therapy is the side-effects that they can inflict on normal cells. For a very long time, scientists are on the search of anti-cancer agents that specifically target tumor cells with no or minimum effects on normal cells. A very recent study indicates that simvastatin, at doses that we had previously shown to induce apoptosis in prostate cancer cells [5], does not compromise cell survival in normal airway epithelial and fibroblast cells, while inducing apoptosis in breast, hepatocellular and lung carcinoma cells [6]. Although this study provides the necessary assurance that simvastatin may be a potential drug for specifically targeting cancer cells for therapy, the molecular mechanisms by which simvastatin induces apoptosis in cancer cells remains to be determined.

Bcl-2-mediated, mitochondria associated cell survival pathway (intrinsic pathway) is one of the major pathways that are targeted for inducing apoptosis in cancer cells. In addition to this, another major pathway that promotes apoptosis in cancer cells is the death receptor-mediated pathway (extrinsic pathway) [7]. Tumor necrosis factor (TNF), TNF-related apoptosis inducing ligand (TRAIL), Fas-ligand (Fas-L), TNF-related factor-1 and 2 (Traf1/2) etc. are some of the key molecules that belong to the extrinsic pathway or death receptor signaling that are known to be de-regulated in cancers [8,9]. While inhibition of Bcl-2-mediated intrinsic pathway leads to the release of cytochrome c from the mitochondria to the cytosol, resulting in the activation of caspases 9 and 3, death receptor-mediated extrinsic pathway involves caspases 10 and 8 in inducing apoptosis [7]. A pre-requisite for the latter is the formation of a death-inducing signaling complex (DISC) between Fas-assciated death domain (FADD) and pro-caspase 8 [10]. Resulting cleavage of pro-caspase 8 to active cleaved caspase 8 leads to the activation of downstream caspases such as caspase 3 [11].

Until recently, docetaxel-based chemotherapy is the only available treatment option for the androgen-insensitive prostate cancer patients and is shown to modestly improve survival [12], marking the first real advance after the identification of therapeutic castration by Charles Huggins in 1941 [13]. Docetaxel (Taxotere®) acts via suppression of microtubule assembly and disassembly, microtubule bundling and inhibition of Bcl-2, leading to apoptosis [14]. However, use of docetaxel is associated with a number of serious side-effects due to yet unknown reasons [15,16]. According to many reports doses of statins, even 50 times higher than the prescribed doses for the treatment of cardiovascular diseases, did not inflict any serious side-effects or toxicity to liver and kidney in men [17-19]. In the current study, we investigated the various mechanisms by which simvastatin induce apoptosis in prostate cancer cells as compared to the known effects of docetaxel treatment. Our study indicates that simvastatin induces apoptosis in prostate cancer cells in *vitro* and prostate tumor xenograft *in vivo* by simultaneously modulating intrinsic and extrinsic apoptotic pathways. These results suggest that simvastatin can be developed as an important drug for the treatment of prostate cancer either alone or in combination with reduced doses of chemotherapeutic drugs such as docetaxel to improve the efficacy and reduce the side-effects.

## Methods

### Cell lines, reagents, and antibodies

Human PC3 and LNCaP cell lines were obtained from ATCC (Manassas, VA) and maintained in DMEM High Glucose (HyClone) with 10% fetal bovine serum (FBS), 100 units/ml penicillin, and 100 μg/ml streptomycin in 5% CO_2_ humidified atmosphere at 37°C. Primary antibodies against pBad, Bcl-2, Bcl-xL, Bim, cleaved caspase 3, cleaved caspase 9, cleaved caspase 8, cytocrocme c, Fas-L, survivin and Traf1 were purchased from Cell Signaling (Boston, MA). Primary antibodies anti-Nme5 was obtained from Abcam (Cambridge, MA/ San Francisco, CA), anti-Trp53inp1 was from R&D (Minneapolis, MN) and anti-β-actin was from Sigma (St Louis, MO). Anti-mouse and anti-rabbit HRP conjugated secondary antibodies were obtained from BioRad (Hercules, CA). Docetaxel and simvastatin were purchased from Sigma (St Louis, MO). Simvastatin was activated in the laboratory using the manufacturer’s instructions.

### Transfections

Adenoviral particles for Bcl-2 and DN-Caspase-9 used for the experiments were obtained from Vector BioLabs (Eagleville, PA). For adeno-infections, PC3 cells were grown until reaching 75 % of confluence in 6-well plates. Next, cells were washed with 1X PBS and 1 ml of DMEM without FBS, supplemented with 10 μg of polybrene was added, followed 5X10^9^ PFU/ml of adeno-Bcl-2 virus and/or 1X10^10^ PFU/ml of andeno-CMV-caspase9 virus. After 48 hours cells were lysed, protein levels were quantified using DL protein assay (Bio-Rad, Hercules, CA) and subjected to western blot analysis.

### Trypan blue viability assessment

In the trypan blue method, cells were grown to confluence in DMEM with 10% FBS. The cells were treated with 25 μM simvastatin, 10 nM docetaxel, or a combination of both in DMEM. After 24h, cells were collected and re-suspended in PBS with 0.4% trypan blue solution. Total cells and trypan blue-stained (i.e., nonviable) cells were counted, and the percentage of nonviable cells was calculated.

### Apoptosis assay

Cytoplasmic histone-associated DNA fragments were quantified by using the Cell Death Detection ELISA^PLUS^ kit (Roche Applied Science, Indianapolis, IN) according to the manufacturer's protocol. Briefly, PC3 cells were plated in 96-well plate at a density of either 10^4^ cells/well. After 24h, the cells were treated with 25 μM simvastatin and/or 10 nM docetaxel for 16h in DMEM containing 10% FBS. Control cells received 0.1% DMSO (vehicle control). Cells were lysed and centrifuged at 200*g* for 10 min, and the collected supernatant was subjected to ELISA. The absorbance was measured at 405 nm (reference wavelength, 492 nm).

### Caspase-9 activity assay

Caspase-9 activity assay were performed using Caspase-Glo® 9 Assay kit according to the manufacturer’s protocol (Promega, Madison, WI). Briefly, PC3 cells were either treated with 25 μM simvastatin, 10 nM docetaxel, and a combination of both, or infected with 5X10^9^ PFU/ml of adeno-Bcl-2 virus and/or 1X10^10^ PFU/ml of adeno-DN-caspase9 virus particles. After plating PC3 cells were plated on a 96-well plate at the density of 2.5x10^4^, 100 μl of Caspase-Glo® 9 Reagent was added to each well and cells were incubated in room temperature for 2.5 h followed by the luminescence measurement using an ELISA plate reader. The data are presented as mean ± S.D.

### *In vivo* nude mouse tumor xenograft model

All animal procedures listed in this article were performed as per the protocol approved by the Institutional Animal Care and Use Committee at the Charlie Norwood Veterans Affairs Medical Center, Augusta, GA (protocol 09-07-011, dated July 10, 2009). PC3 cells were grown to confluence in 250-ml flasks. Cells were re-suspended in PBS to a concentration of 10^6^/ml. Cell suspension (1 μl) was injected subcutaneously in 6- to 8-week-old nude mice (athymic nude mice; Harlan, Indianapolis, IN). The mice were subjected to intraperitoneal injections of simvastatin at a dose of 2 mg/kg body weight every 12h for 2 weeks. The respective controls were injected intraperitoneally with 0.9% saline every 12h. Mice were sacrificed on day 14, and tumors were dissected, weighed, and snap frozen using dry ice for further processing to use on western or qRT-PCR.

### Terminal deoxynucleotidyl transferase-mediated dUTP nick end labeling (TUNEL) assay

The TUNEL assay for *in situ* detection of apoptosis was performed by using the ApopTag® Fluorescein In Situ Apoptosis detection kit (Millipore, MA) according to the manufacturer’s instructions. Cells were plated in 24-well flat bottom plates at a density of 1 x 10^5^ cells/well and treated with 25 μM simvastatin, 10nM docetaxel or a combination of both for 24h. Following treatments, cells were fixed in 2% paraformaldehyde at 4°C for 30 min. Fixed cells were then permeabilized in 0.1% Triton X-100 and labeled with fluorescein 12-dUTP using terminal deoxynucleotidyl transferase. Nuclei were counterstained with DAPI. Frozen nude mouse prostate tumor (PC3) xenograft sections were also processed accordingly. Cells/tissue sections were analyzed for apoptotic cells with localized green fluorescence using an inverted fluorescence microscope (Zeiss Axiovert100M, Carl Zeiss, Germany).

### QReal-time PCR arrays

PC3 cells were grown until reaching 75% of confluence in 6-well plates and subjected to RNA isolation, followed cDNA synthesis and qPCR quantification. Briefly, cells were lysed and RNA was isolated according to manufacturer’s protocol using RNAese Mini Plus Kit (Qiagen, Valencia, CA). Next, 25 μl of cDNA was produced by RT^2^ First Strand Kit (SABioscience, Frederick, MD), mixed with qPCR SyberGreen master mix and loaded into Human Apoptosis RT^2^ Profiler PCR Array plate (SABiosciences, Frederick, MD). Reading was completed in Eppendorf Mastercycler realplex 2 instrument.

### Western blot analysis

PC3 cells were cultured in 6-well plates to reach a monolayer. At that point, the cells were treated with 25 μM simvastatin and/or 10 nM docetaxel in DMEM supplemented with 10% FBS. Control cells received 0.1% of DMSO. Whole cell lysates were prepared using lysis buffer [50 mM Tris–HCl (pH=7.4), 1% TritonX-100, 150mM NaCl, 1mM EDTA, 2mM Na_3_VO_4_, and 1X Complete protease inhibitors (Roche Applied Science, Indianapolis, IN)]. Tumors isolated from mice with C53BL/6 background treated with 2mg/kg simvastatin for 11 days, were first snap frozen in liquid nitrogen and then pulverized with mortar and piston. Next, tissues lysates were prepared using lysis buffer. The protein concentration was measured by the DL protein assay (Bio-Rad, Hercules, CA). 60 μg/μl of protein was subjected to western blot analysis according to standard Laemmli’s method.

### Statistical analysis

Mean activities were calculated from 3–5 independent experiments done at least in triplicates. The Student’s two-tailed t test was used to determine significant differences between treatment and control values.

## Results

### Simvastatin induces cell death and apoptosis in prostate cancer cells

**Figure 1:**
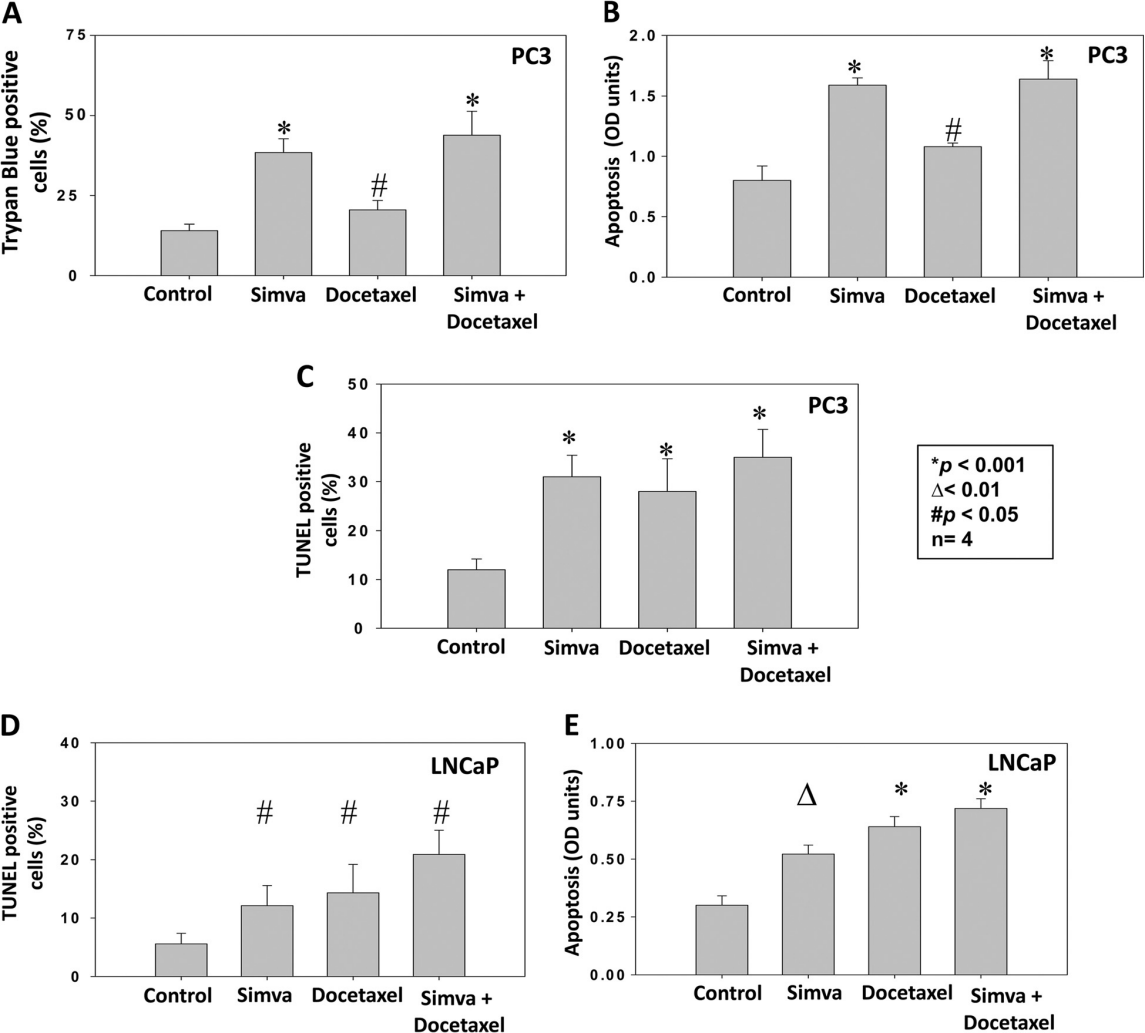
**Simvastatin induces cell death and apoptosis in prostate cancer cells. **(**A**) Bar graph showing trypan blue positive PC3 cells treated with control saline, simvastatin, docetaxel or a combination of simvastatin and docetaxel for 12 h. (**B**) Bar graph showing apoptosis in PC3 cells treated with control saline, simvastatin, docetaxel or a combination of simvastatin and docetaxel for 24 h as measured calorimetrically. (**C**) Bar graph showing quantification of TUNEL positive PC3 cells treated with control saline, simvastatin, docetaxel or a combination of simvastatin and docetaxel for 24 h. (**D**) Bar graph showing quantification of TUNEL positive LNCaP cells treated with control saline, simvastatin, docetaxel or a combination of simvastatin and docetaxel for 24 h. (**B**) Bar graph showing apoptosis in LNCaP cells treated with control saline, simvastatin, docetaxel or a combination of simvastatin and docetaxel for 24 h. The data are presented as mean ± SD (n=4 of quadruplicate experiments).

Since simvastatin inhibited activity of the cell survival kinase Akt [5], we studied whether treatment with simvastatin will compromise cell survival and induce apoptosis in prostate cancer (PC3 and LNCaP) cells. A trypan-blue dye based study indicated that treatment with 25 μM simvastatin induced >2-fold increase in PC3 cell death in 24h, compared to saline treated controls (*p*<0.001) (Figure 1A). The effect of simvastatin on PC3 cell death was higher than the effect of 10 nM docetaxel, a currently approved drug for prostate cancer therapy. Interestingly, a combination of simvastatin and docetaxel further enhanced PC3 cell death by another fold, compared to simvastatin treated cells (*p*<0.01) and 3-fold higher compared to saline treated controls (*p*<0.001) (Figure 1A). We next performed apoptosis assay using a method that measures the cytoplasmic histone-associated DNA fragments. Our data confirmed that both simvastatin and docetaxel significantly induced apoptosis in PC3 cells (*p*<0.001 and *p*<0.05, respectively) (Figure 1B). However, although a trend was noted, the combined effect of simvastatin and docetaxel on the apoptosis of PC3 cells was not observed. In order to further confirm our data, we performed TUNEL assay to assess DNA fragmentation as a late event in the process of apoptosis in PC3 cells. Our TUNEL staining data further confirmed that while simvastatin and docetaxel independently induced apoptosis in PC3 cells (*p*<0.001 and *p*<0.05, respectively), a combination of these drugs exhibited a modest increase in apoptosis compared to each of these drugs alone (Figure 1C). We went on to determine whether the effects of simvastatin on apoptosis are also applicable to androgen-responsive LNCaP cells. Our data indicated that confirmed that both simvastatin and docetaxel induced apoptosis in LNCaP cells as evidenced from the TUNEL staining (*p*<0.05) and (*p*<0.01 and *p*<0.001, respectively) apoptosis assays (Figure 1D and E). Overall, our study demonstrates that simvastatin induces apoptosis in prostate cancer cells.

### Simvastatin inhibits Bcl-2-mediated intrinsic pathway in prostate cancer cells

**Figure 2:**
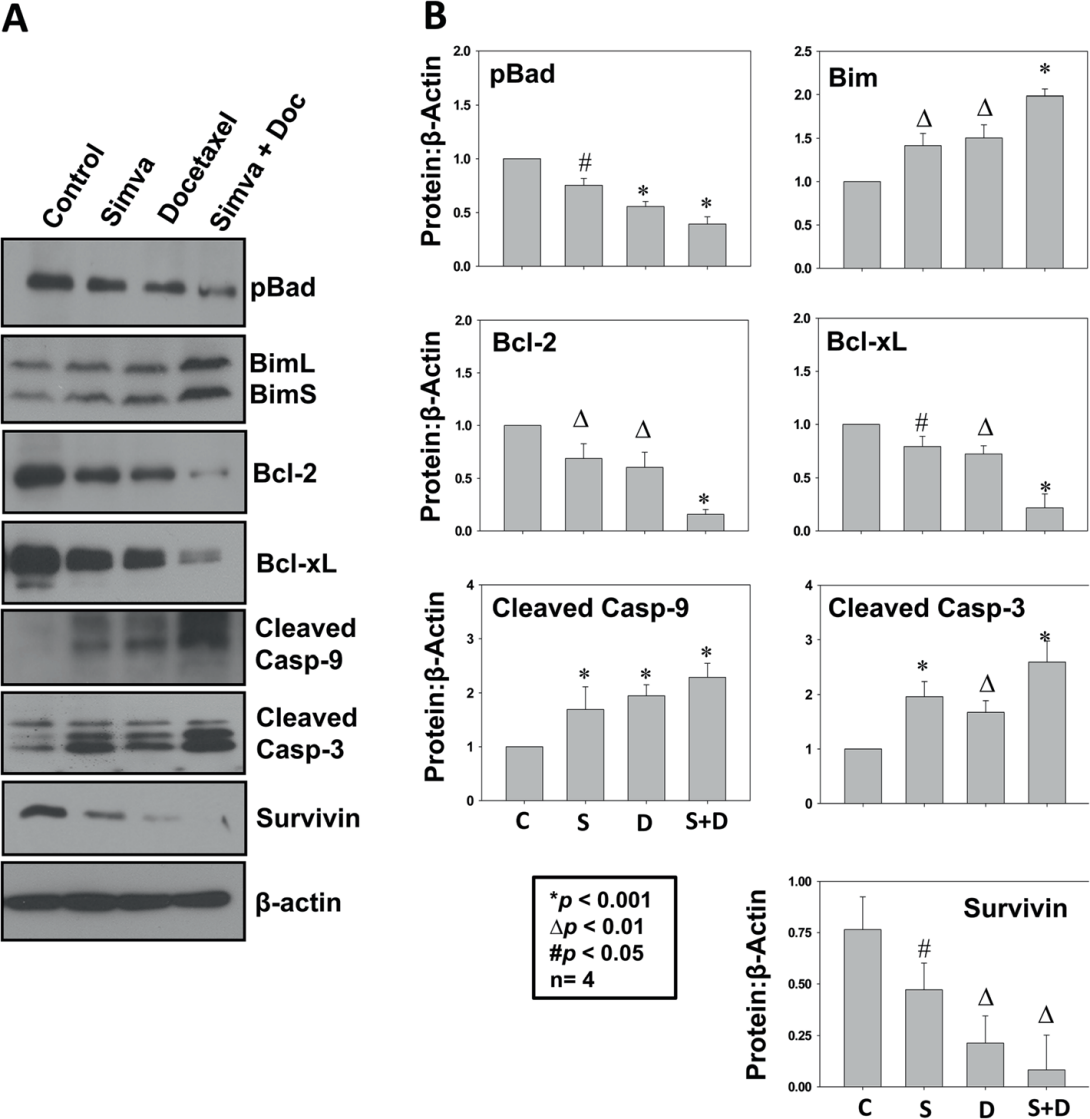
**Simvastatin inhibits Bcl-2- and Bcl-xL-mediated cell survival pathway in prostate cancer cells.** (**A**) Western blots showing reduced phosphorylation of Bad, reduced protein expression of Bcl-2 and Bcl-xL as well as increased protein expression of BimL/BimS, cleaved caspase 9 and cleaved caspase 3 after 24 h treatment with simvastatin, docetaxel or a combination of both, compared to saline treated control (**B**) Bar graph showing quantification of the above data by densitometry analysis normalized to β-actin. The data are presented as mean ± SD (n=4 of quadruplicate experiments).

Akt is known to modulate Bcl-2-mediated cell survival pathway via phosphorylation of Bcl-2-associated death promoter (Bad). We determined whether simvastatin treatment inhibited Bcl-2-mediated cell survival pathway in prostate cancer cells. Our data indicated that treatment with simvastatin significantly impaired phosphorylation of Bad (*p*<0.05), decreased protein expression of Bcl-2 and Bcl-xL (*p*<0.01 and *p*<0.05, respectively) as well as increased protein levels of BimL/BimS (*p*<0.01), cleaved caspase 9 and cleaved caspase 3 (*p*<0.001) (Figures 2A and B). These effects were similar to the treatment of prostate cancer cells with docetaxel. Eventhough a synergistic effect on the protein expression of Bcl-2 and Bcl-xL was seen in prostate cancer cells with combined treatment of simvastatin and docetaxel, a net significant additive effect on the final products of intrinsic pathway such as cleaved caspase 3 and cleaved caspase 9 was not observed (Figure 2A and B). Together, our results indicate that inhibition of Bcl-2-dependent intrinsic pathway is involved in the simvastatin-mediated effects of PC3 cells.

### Simvastatin induces apoptosis in prostate tumor xenografts via inhibition of intrinsic cell survival pathway

**Figure 3:**
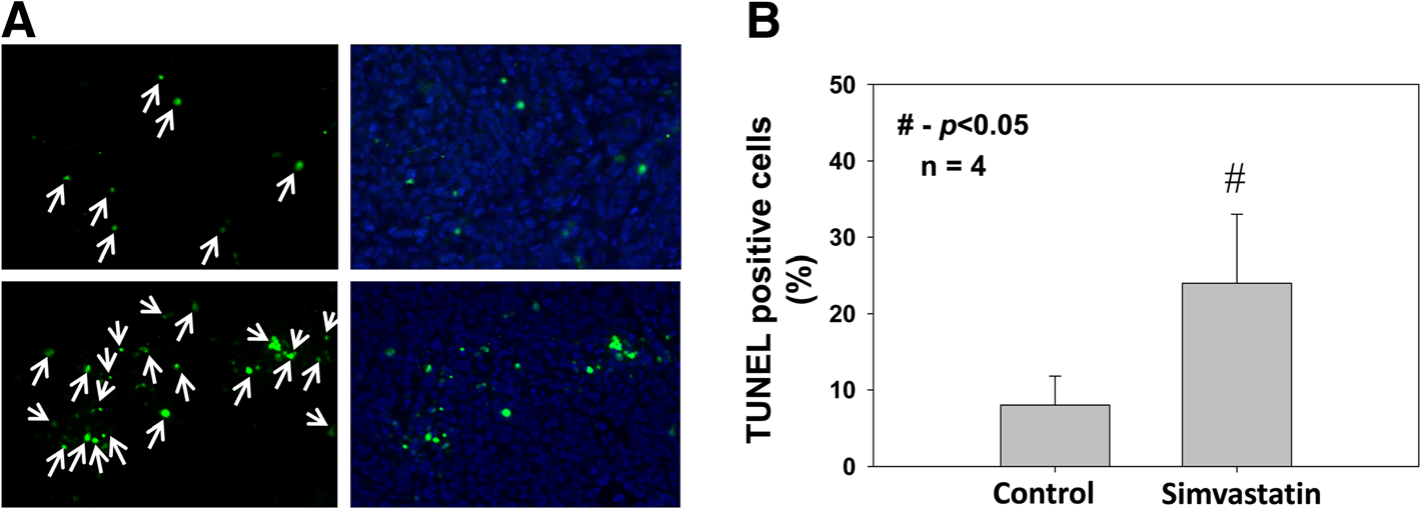
**Simvastatin induces cell death and apoptosis in prostate tumor xenografts. **(**A**) Pictures showing TUNEL staining of PC3 cell nude mice tumor xenogafts treated with control saline or simvastatin for 14 days. (**B**) Bar graph showing quantification of the TUNEL positive PC3 cells in tumor xenogafts treated with control saline or simvastatin for 14 days. The data are presented as mean ± SD (n=4 of quadruplicate experiments).

**Figure 4:**
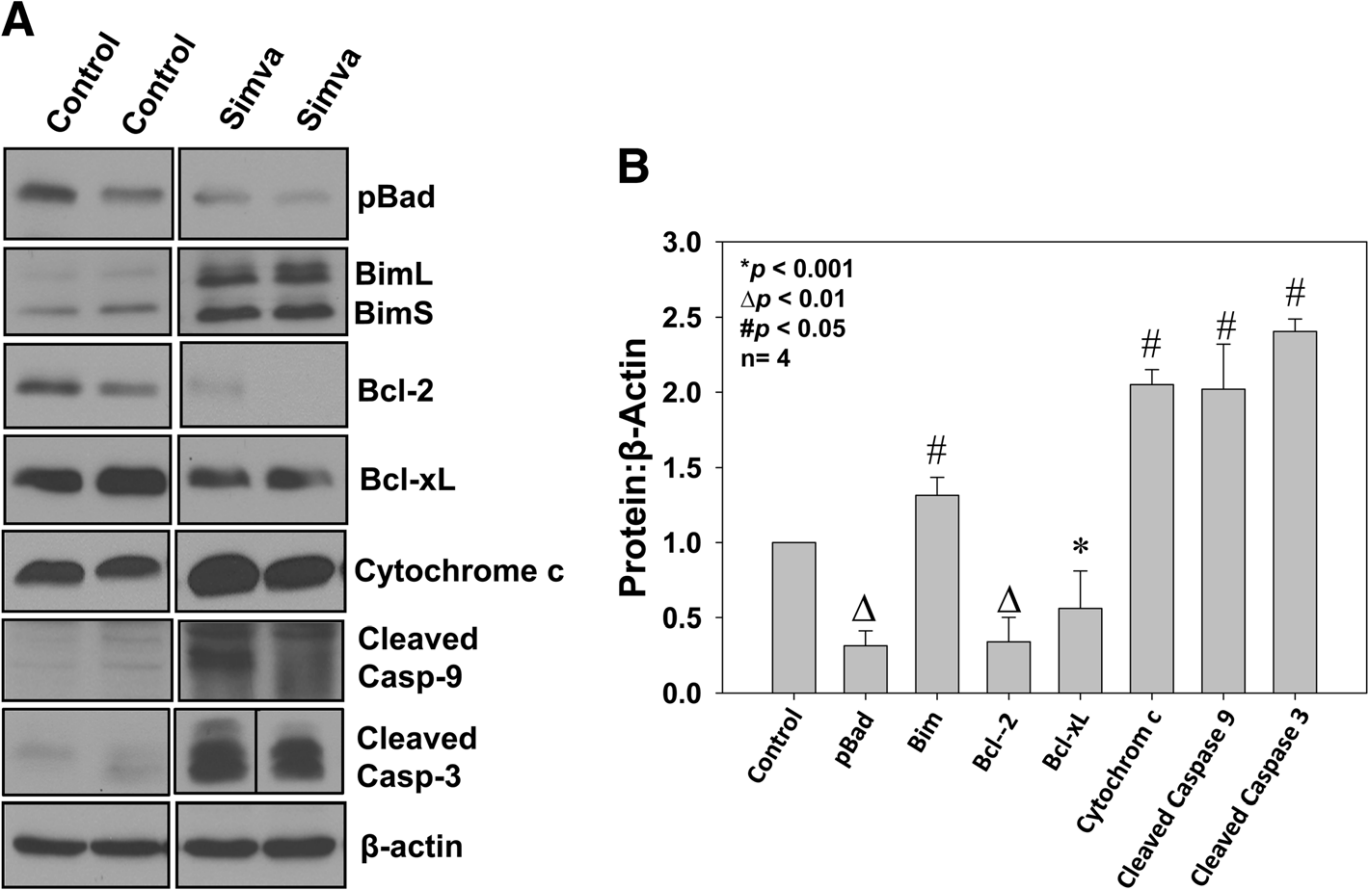
**Simvastatin induces apoptosis in prostate tumor cells *in vivo *via inhibition of intrinsic cell survival pathway. **(**A**) Western blots showing reduced phosphorylation of Bad, reduced protein expression of Bcl-2 and Bcl-xL, increased release of cytochrome-c from the mitochondria as well as increased protein expression of BimL/BimS, cleaved caspase 9 and cleaved caspase 3 with 14 day simvastatin treatment, compared to saline control in PC3 cell nude mice tumor xenogafts (**B**) Bar graph showing quantification of the above data by densitometry analysis normalized to β-actin. The data are presented as mean ± SD (n=4 of quadruplicate experiments).

We next determined whether simvastatin treatment has any effect on prostate tumor cell survival *in vivo*. In order to do this, frozen sections of PC3 tumor xenografts from athymic nude mice were subjected to TUNEL assay. Our data indicated that treatment with simvastatin in nude mice (2mg/kg body weight/12 hours, intra-peritoneally) significantly enhanced apoptosis in tumors compared to saline treated controls by >2-fold (*p*<0.05) (Figure 3A and B). Western analysis of the tumor lysates indicated that, similar to prostate cancer cells *in vitro*, treatment with simvastatin significantly impaired phosphorylation of Bad (*p*<0.01), decreased protein levels of Bcl-2 and Bcl-xL (*p*<0.01 and *p*<0.001, respectively), increased release of cytochrome C from the mitochondria to cytosol (*p*<0.05 ) as well as increased protein expressions of BimL/BimS, cleaved caspase 9 and cleaved caspase 3 (*p*<0.05), compared to saline treated controls (Figures 4A and B).

### Prostate cancer cells over-expressing Bcl-2 and/or DN-Caspase 9 are not resistant to simvastatin induced apoptosis

**Figure 5:**
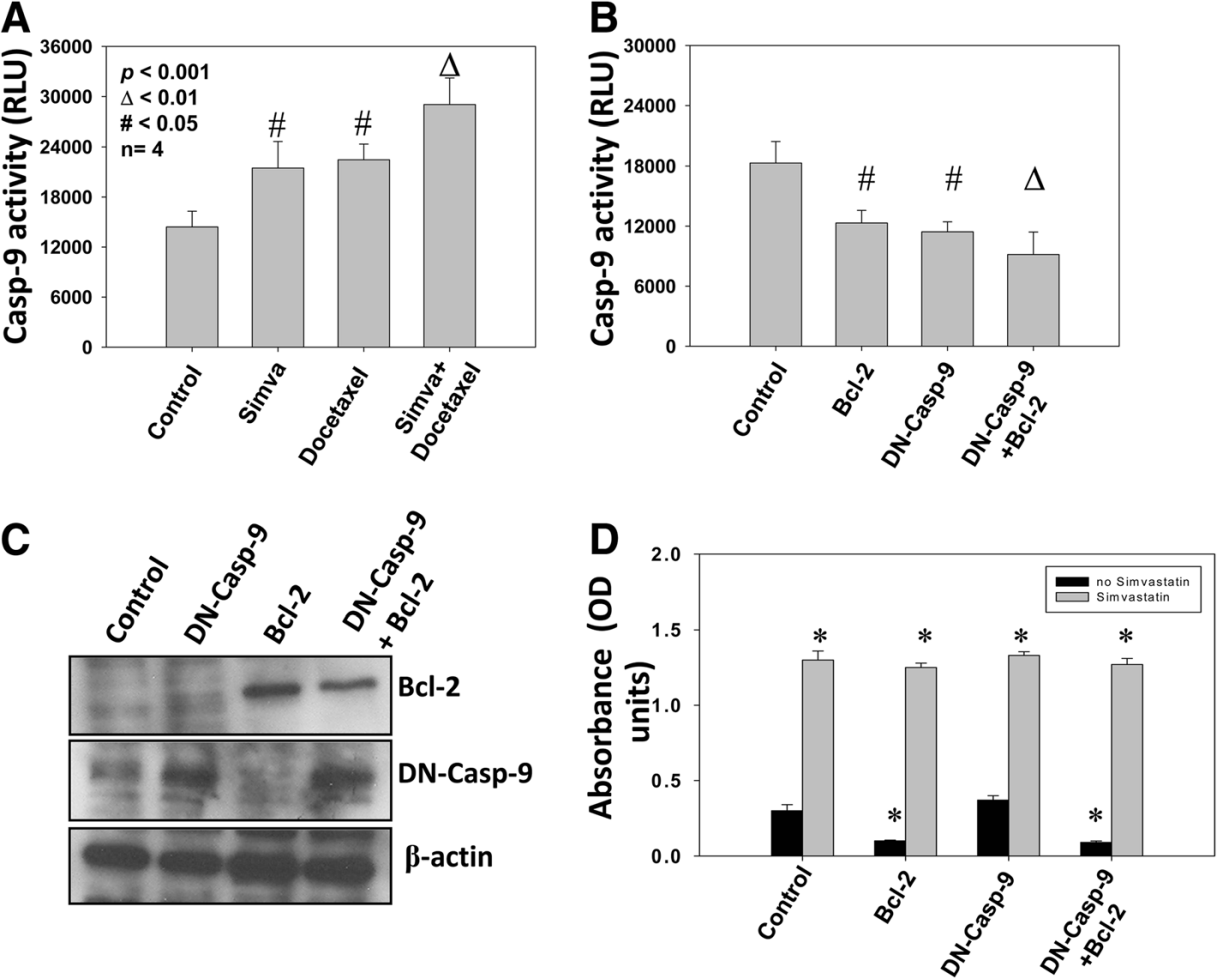
**Over-expression with Bcl-2 or DN-caspase 9 does not rescue the simvastatin induces apoptosis in prostate tumor cells. **(**A**) Bar graph showing caspase-9 activity in PC3 cells after 24 h treatment with simvastatin, docetaxel or a combination of both, compared to saline treated control. (**B**) Bar graph showing caspase-9 activity in PC3 cells over-expressing Bcl-2, DN-caspase 9 and both, compared to vector control in the presence and absence of 24h treatment with simvastatin. (**C**) Western blots showing protein expression of Bcl-2 and caspase-9 in PC3 cells over-expressing Bcl-2, DN-caspase 9 and both. (**D**) Bar graph showing apoptosis of PC3 cells over-expressing Bcl-2, DN-caspase 9 and both, compared to vector control in the presence and absence of 24h treatment with simvastatin. The data are presented as mean ± SD (n=4 of quadruplicate experiments).

We first determined the effect of simvastatin and docetaxel on caspase 9 enzymatic activity in PC3 cells. Our data show that both simvastatin and docetaxel significantly induced caspase-9 activity in PC3 cells (*p*<0.05) with a combined effect when simvastatin and docetaxel are used together (*p*<0.01) (Figure 5A). To determine whether Bcl-2-mediated intrinsic cell survival pathway is the solely affected pathway in prostate cancer cells, we performed rescue experiments by treating PC3 cells over expressing either Bcl-2 or DN-caspase 9 (cleavage resistant) or a combination of both, along with control cells over expressing plasmid vectors. Changes in enzymatic activity of caspase 9 were also confirned with Bcl-2 and DN-caspase 9 plasmid expression in PC3 cells (Figure 5B). Our data indicated that over-expression with Bcl-2, but not caspase-9, enhanced cell survival in PC3 cells (*p*<0.001) (Figure 5C and D) as measured by the ELISA-based apoptosis assay kit. However, these cells were not resistant to treatment with simvastatin. No significant inhibition of simvastatin-induced apoptosis was observed by over-expressing PC3 cells with Bcl-2, DN-caspase 9 or a combination of both, compared to vector only expressing controls (Figure 5C and D). Our data indicate that pathways in addition to intrinsic pathway are involved in simvastatin-induced apoptosis in PC3 cells.

### Simvastatin modulates expression of genes involved in the death receptor-mediated apoptotic pathway in prostate cancer cells

**Figure 6:**
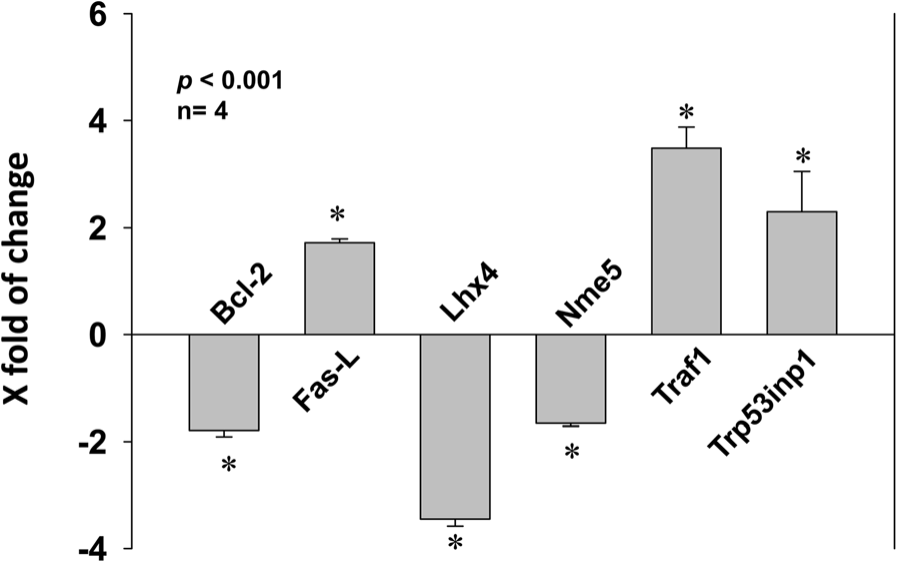
**Simvastatin modulates expression of genes in PC3 cells involved in the extrinsic pathway regulating apoptosis. **Bar graph showing changes in the mRNA levels of genes such as Bcl-2, Fas-L, Lhx4, Nme5, Traf1 and Trp53inp1with 24h simvastatin treatment normalized to multiple housekeeping genes. The data are presented as mean ± SD (n=4 of quadruplicate experiments).

Since over-expression of PC3 cells with Bcl-2 and/or DN-caspase 9 did not rescue from simvastatin-induced apoptosis, we hypothesized that pathways other than intrinsic cell survival pathway may also be inhibited by simvastatin. To study this, we performed Real-Time qPCR-based gene arrays specific for genes involved in the regulation of cell survival and apoptosis. From our gene array analysis, we identified several candidate genes that are likely involved in the simvastatin-induced apoptosis in PC3 cells (Table 1). Some of the candidate genes whose expressions were significantly modulated by statin in PC3 cells included Bcl-2, Fas-L, Lhx4, Nme5, Traf1 and Trp53inp1 (*p*<0.001), many of them involved in the extrinsic death receptor-mediated apoptosis pathway (Figure 6).

**Table 1:** Genes modulated by simvastatin in PC3 cells as identified by qRT-PCR arrays

**GeneBank**	**Symbol**	**Description**	**Change (X fold)**
NM_030693	Atf5	Activating transcription factor 5	2.0↓
NM_009741	Bcl2	B-cell leukemia/lymphoma 2	2.0↓
NM_009743	Bcl2l1/2	Bcl2-like 1 and 2	1.7↓
NM_013479	Bcl2l10	Bcl2-like 10	2.0↓
NM_008670	Birc1a	Baculoviral IAP repeat-containing 1a	2.4↑
NM_007464	Birc3	Baculoviral IAP repeat-containing 3	1.6↑
NM_009689	Birc5	Baculoviral IAP repeat-containing 5	3.0↑
NM_009807	Casp1	Caspase 1	2.8↑
NM_007702	Cidea	Cell death-inducing DNA fragmentation factor, alpha subunit-like effector A	1.6↑
NM_010015	Dad1	Defender against cell death 1	2.5↓
NM_010175	Fadd	Fas (TNFRSF6)-associated via death domain	2.0↑
NM_010177	Fasl	Fas ligand (TNF superfamily, member 6)	1.9↑
NM_010548	Il10	Interleukin 10	1.6↑
NM_010712	Lhx4	LIM homeobox protein 4	3.3↓
NM_080637	Nme5	Non-metastatic cells 5, protein expressed in (nucleoside-diphosphate kinase)	1.8↓
NM_030152	Nol3	Nucleolar protein 3 (apoptosis repressor with CARD domain)	1.6↓
NM_023258	Pycard	PYD and CARD domain containing	2.0↓
NM_013693	Tnf	Tumor necrosis factor	3.2↑
NM_020275	Tnfrsf10b	Tumor necrosis factor receptor superfamily, member 10b	1.6↑
NM_011611	Cd40	CD40 antigen	3.5↑
NM_009425	Tnfsf10	Tumor necrosis factor (ligand) superfamily, member 10	1.6↑
NM_011617	Cd70	CD70 antigen	3.3↑
NM_009421	Traf1	Tnf receptor-associated factor 1	4.0↑
NM_021897	Trp53inp1	Transformation related protein 53 inducible nuclear protein 1	3.2↑

### Simvastatin, but not docetaxel is involved in the activation of Fas-L mediated extrinsic pathway in prostate cancer cells and tumor xenografts

**Figure 7:**
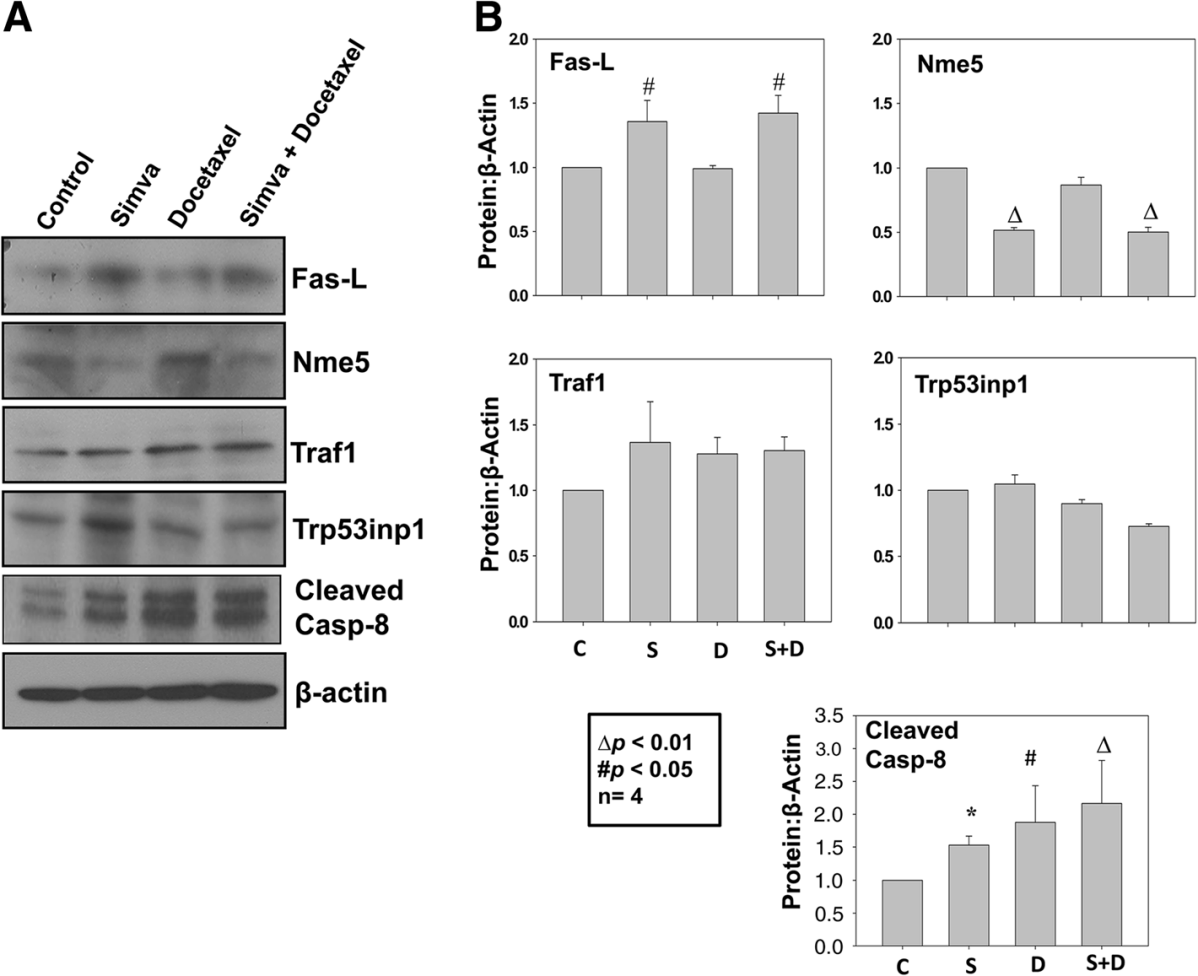
**Simvastatin modulates expression of pro-apoptotic extrinsic pathway proteins in PC3 cells. **(**A**) Western blots showing protein expression of Fas-L, Nme5, Traf1, cleaved caspase-8 and Trp53inp1 in PC3 cells treated with simvastatin or docetaxel, compared to control saline treated cells. (**B**) Bar graph showing quantification of the above data by densitometry analysis normalized to β-actin. Increase in the protein expression of Traf1, Fas-L and Trp53np1 as well as decreased protein expression of Bcl-2 and Nme5 was observed with simvastatin treatment, but not with docetaxel, compared to control. The data are presented as mean ± SD (n=4 of quadruplicate experiments).

To investigate whether these genes were regulated by simvastatin in prostate cancer cells at the protein level, we performed western analysis of PC3 cells treated with either saline control or simvastatin. Our data showed that treatment with simvastatin while significantly increased protein expression of pro-apoptotic Fas-L (*p*<0.05), it inhibited expression of pro-survival protein Nme5 (*p*<0.01) (Figure 7A and B). Although a trend towards increased protein expression of Traf1 was observed with simvastatin treatment in PC3 cells lysates, this was however not significant (Figure 7B). In any case, treatment with docetaxel did not have any effect on the expression of proteins involved in the extrinsic pathway involving Fas/Fas-L. Interestingly, we did observe some changes in cleaved caspase 8 protein levels with both simvastatin (*p*<0.001) and docetaxel treatment (*p*<0.05), suggesting that docetaxel may also be involved in the regulation of extrinsic pathway through a Fas/Fas-L independent mechanism (Figure 7B).

**Figure 8:**
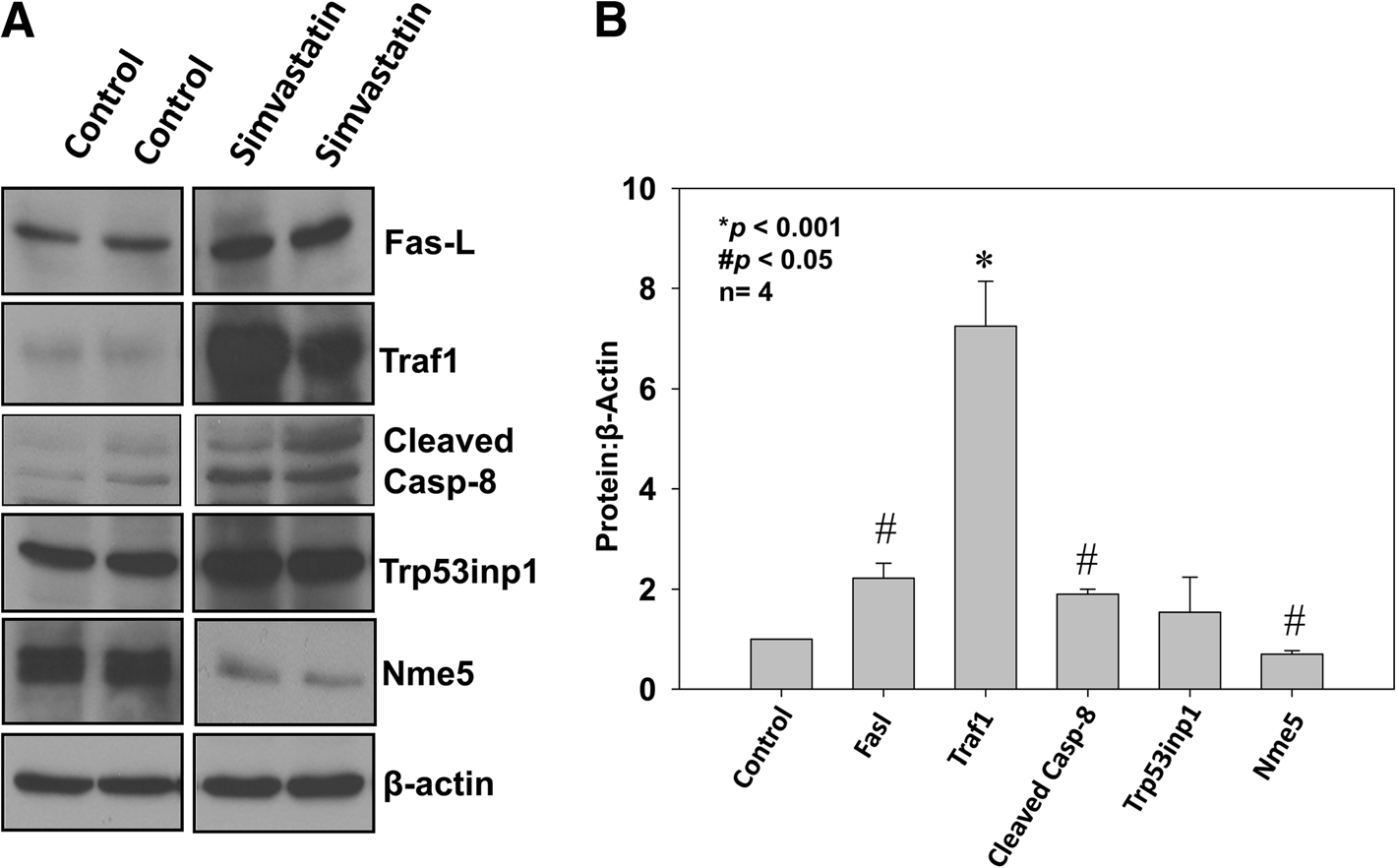
**Simvastatin modulates expression of Fas-L, Traf1 and cleaved caspase 8 in prostate tumor xenografts. **(**A**) Western blots showing protein expression of Fas-L, Nme5, Traf1, cleaved caspase 8 and Trp53inp1 in PC3 cell tumor xenografts treated with simvastatin, compared to control saline treated tumors. (**B**) Bar graph showing quantification of the above data by densitometry analysis normalized to β-actin. Increased protein expression of Traf1, Fas-L, Trp53np1 and cleaved caspase-8 as well as decreased protein expression of Bcl-2 and Nme5 with simvastatin treatment, but not with docetaxel, compared to control is evident. The data are presented as mean ± SD (n=4 of quadruplicate experiments).

Using Western analysis of the tumor lysates, we next determined whether simvastatin has effect on extrinsic pathway components in PC3 tumor xenografts *in vivo*. Our data indicated that while protein levels of Fas-L and Traf1 was significantly increased in PC3 tumors treated with simvastatin, compared to saline treated controls (*p*<0.05 and *p*<0.001, respectively), protein expression of Nme5 was significantly reduced (*p*<0.05) (Figure 8A and B). Further analysis of tumor cell lysates revealed that protein expression of cleaved caspase 8, a molecule involved in the extrinsic pathway downstream of activated caspase 10 was significantly increased in tumor xenografts treated with simvastatin, compared to saline treated controls (*p*<0.01) (Figure 8A and B).

## Discussion

**Figure 9:**
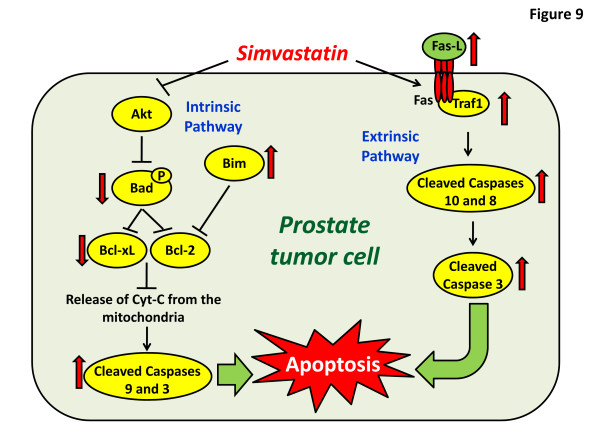
Working hypothesis on the mechanisms by which simvastatin induces apoptosis in prostate cancer cells involving both intrinsic and extrinsic pathways.

Many recent studies [1], including ours [5] show that statins are beneficial as anti-cancer agents via inhibition of prostate cancer cell functions *in vivo* such as proliferation, cell survival, cell migration and colony formation etc. In this study, we have shown that treatment of prostate cancer cells with simvastatin *in vitro* and mice bearing prostate tumor xenograft *in vivo* significantly induce apoptosis in prostate cancer cells. Simvastatin-mediated effects on prostate cancer cell viability and apoptosis was superior to the effects of docetaxel, a currently approved drug for the chemotherapy of prostate cancer patients. Although a combined effect on prostate cancer cell viability was observed by treating simvastatin along with docetaxel, this effect was not observed in assays specific for apoptosis such as TUNEL and cytoplasmic histone-associated DNA fragment assays. While Bcl-2-mediated mitochondria-associated intrinsic cell survival pathway was significantly inhibited in PC3 cells and tumor xenografts by simvastatin treatment, over-expression of PC3 cells with Bcl-2 and/or dominant negative caspase 9 did not reverse the simvastatin-mediated PC3 cell apoptosis. While simvastatin treatment reduced the expression of phosphorylated-Bad, Bcl-2, Bcl-xL and survivin in PC3 cells, it resulted in increased protein expression of Bim, cleaved caspases 9 and 3, with an increased effect in the presence of docetaxel. Modulation of Bcl-2-pathway with simvastatin was also observed in PC3 tumor lysates. Gene arrays followed by western analysis of PC3 cell and tumor lysates treated with simvastatin identified several genes involved in the extrinsic death-receptor apoptosis pathway modulated by simvastatin, but not with docetaxel, such as tumor necrosis factor (TNF), Fas-L, Traf1 and cleaved caspase 8, along with other genes such as Lhx4, Nme5 and Trp53inp1, which are novel, yet unknown regulators of cell survival and apoptosis in prostate cancer cells. Altogether, our results have demonstrated that simvastatin induces apoptosis in prostate cancer cells via simultaneous modulation of intrinsic and extrinsic pathways (Figure 9).

Because of its ‘crossroad’ role in multiple essential signaling pathways in cancer cell maintenance, and its enhanced expression and/or activation in multiple cancer cells as compared to normal, Akt kinase is being actively pursued as a novel target for cancer therapy [20-23]. However, since Akt is essential for many normal cell functions [24-26], cell survival in particular, targeting Akt for cancer therapy is a bottle neck due to the serious side-effects associated with it. This asks for novel therapies that can inflict a significant but selective effect on cancer cells in inhibiting pathways like Akt without affecting the normal functioning of extra-tumor tissues. Many recently published reports suggest that statins, at certain higher doses, can be a selective and very efficient drug to treat cancers without inflicting any major side-effects [17-19]. We previously showed that simvastatin, at a dose ~5 times higher than the therapeutic dose prescribed for the treatment of cardiovascular diseases, significantly inhibited Akt activity in PC3 tumor cells and prostate tumor xenograft growth *in vivo*[5]. Another recent report indicated that at similar doses, simvastatin induced apoptosis in breast cancer cells, but not in normal airway epithelial cells or fibroblasts [6]. Thus, the ability of simvastatin to selectively inhibit Akt activity and induce apoptosis in prostate cancer cells without affecting the normal cells makes it an attractive candidate for drug re-purposing for cancer therapy.

Many of the effects of simvastatin on prostate cancer cell apoptosis can be credited to its ability to inhibit Akt activity. Akt is known to enhance the intrinsic mitochondria-associated cell survival pathway in cancer cells via increased phosphorylation of Bad and enhanced expression of Bcl-2 and Bcl-xL [7]. Upon inhibition of Akt by simvastatin in PC3 cells, we saw reduced phosphorylation of Bad, decreased expression of Bcl-2 and Bcl-xL, associated with increased expression of Bim as well as cleaved caspases 9 and 3. Activated caspase 3 is expected to further cleave PARP in inducing apoptosis [7]. Inhibition of Bcl-2-mediated pathway by statins has also been shown by other labs in multiple cancer types [6,27,28]. However, our attempt to rescue the PC3 cells from apoptosis by re-constituting the Bcl-2 pathway by over-expressing PC3 cells with Bcl-2 and/or DN-caspase 9 did not reverse the simvastatin-induced apoptosis. This suggested that pathways other than intrinsic survival pathway are involved in simvastatin-induced apoptosis in prostate cancer cells.

On the other end, gene arrays as well as western analysis of cell and tumor lysates identified a number of novel candidates that are involved in the simvastatin-induced apoptosis in prostate cancer cells. One of the pro-survival proteins that were found to be less expressed in simvastatin-treated PC3 cells was survivin, which is also associated with mitochondria-associated cell survival pathway. Survivin is highly expressed in many cancer cells [29], including prostate cancer cells [30,31]. Regulation of survivin expression in multiple experimental models has been linked to increase in Akt activity [32]. In prostate cancer cells, survivin expression has been shown to be regulated by IGF-1 stimulated Akt-mTOR signaling [33], which Is impaired upon simvastatin treatment [5]. A second pro-survival molecule that is significantly less expressed in simvastatin-treated PC3 cells is non-metastatic cells 5 (Nme5). Nme5, also known as the inhibitor of p53-induced apoptosis-beta (IPIA-beta) is known to confer protection from cell death by Bax and alter the cellular levels of several anti-oxidant enzymes such as Gpx5 [34]. A third molecule that was significantly less expressed in PC3 cells with simvastatin treatment was Lhx4, a molecule abundantly expressed in many cancers [35,36], but exact function is yet to be determined. Other molecules that are de-regulated with simvastatin-treatment in PC3 cells include CD70 (TNFRSF7), CD40, caspase-1, Trp53inp1 and TNFRSF10b etc. (Table 1).

Another mechanism by which apoptosis can be triggered in cancer cells is via signaling by death receptor members that belong to the tumor necrosis factor receptor super-family [37]. Among the eight members of the death receptor family, most common are the TNF receptor 1 (TNFR1 or DR1) and Fas (CD95 or DR2) [7]. Our gene array results indicated an increase in TNF and Fas-L in prostate cancer cells, which are ligands for TNFR1 and Fas, respectively, with simvastation treatment. Furthermore, increase in the expression of other molecules associated with the Fas receptor such as Traf1 and Fas (TNFRSF6)-associated via death domain (FADD) leading to activation of caspase-8 was also observed in PC3 cells and/or tumor lysates with simvastatin treatment. In order to induce apoptosis, TNF and Fas-L utilizes two different death receptor signaling complexes. Fas-L-mediated mechanism comprises the death-inducing signaling complexes (DISCs) that are formed at the CD95 or Fas receptor between Fas-assciated death domain (FADD) and pro-caspases 10 and 8 [10]. Formation of DISC results in the activation of caspases 10 and 8, which place a central role in the transduction of death signal [10,38]. TNF induces apoptosis via a mechanism different from Fas-induced cell death involving two different signaling complexes [39]. Complex-I is formed at the membrane and comprises TNF, TNFR1, receptor-interacting protein (RIP), TNFR-associated death domain (TRADD), TNFR-associated factors 1 and 2 (Traf-1/2) etc. and acts through a JNK-dependent mechanism. Complex-II, also known as traddosome, consists of FADD and caspase 8, which are absent in complex-I [11]. An increase in the levels of cleaved caspase 8 in the PC3 tumor lysates from simvastation-treated mice indicate that one or both of the Fas-L and TNF-mediated death-receptor signaling pathway is involved in simvastatin-induced apoptosis in prostate cancer cells.

## Conclusions

In conclusion, our results have demonstrated that treatment with simvastatin induces apoptosis in prostate cancer cells *in vitro* and tumor xenograft *in vivo* via simultaneous modulation of mitochondria-associated intrinsic pathway that comprises Bcl-2, Bcl-xL and caspases 9 and 3 as well as Fas-L and TNF-dependent extrinsic death receptor pathway involving caspase-8. Our study reinforces the rationale of selective pharmacologic inhibition of prostate cancer cell survival using statins and suggests re-purposing of lipophilic statins such as simvastatin for prostate cancer therapy in humans. Alternatively, statins may also be used in combination with other cytotoxic agents such as docetaxel to improve the drug efficacy and reduce the side-effects.

## Abbreviations

Bcl-2: B-Cell lymphoma-2; Bcl-xL: B-Cell lymphoma extra-large; DR: Death receptor; DISC: Death-inducing signaling complex; EDTA: Ethylenediaminetetraacetic acid; FADD: Fas-assciated death domain; Lhx4: LIM homeobox protein-4; Nme5: Non-metastatic cells 5; RIP: Receptor-interacting protein; TNF: Tumor Necrosis factor; TNFRSF: Tumor Necrosis factor receptor superfamily; TRAIL: TNF-related apoptosis inducing ligand; TRADD: TNFR-associated death domain; Traf1/2: TNF-related factor 1 and 2; TUNEL: Terminal deoxynucleotidyl transferase-mediated dUTP nick end labeling.

## Competing interests

Authors declare no conflict of interests.

## Authors’ contributions

Experiments were conceived and designed by AG, STK and PRS. AG, STK, BA, AA and SM were involved in performing experiments and analyzing the data. Manuscript was written by PRS, AG and STK. All authors revised the manuscript for important intellectual content. All authors read and approved the final manuscript.

## Pre-publication history

The pre-publication history for this paper can be accessed here:

http://www.biomedcentral.com/1471-2407/12/409/prepub
